# Zileuton, a 5-Lipoxygenase Inhibitor, Exerts Anti-Angiogenic Effect by Inducing Apoptosis of HUVEC via BK Channel Activation

**DOI:** 10.3390/cells8101182

**Published:** 2019-09-30

**Authors:** Hyun-Joung Lim, Jinbong Park, Jae-Young Um, Sang-Seob Lee, Hyun-Jeong Kwak

**Affiliations:** 1 Division of Brain Diseases, Center for Biomedical Sciences, Korea National Institute of Health, 187 Osongsaengmyung2-ro, Osong-eup, Heungdeok-gu, Cheongju-si, Chungcheongbuk-do 28159, Korea; hjlim1121@hanmail.net; 2 Department of Pharmacology, College of Korean Medicine, Kyung Hee University, Dongdaemun-Gu, Seoul 02447, Korea; thejinbong@khu.ac.kr (J.P.); jyum@khu.ac.kr (J.-Y.U.); 3 Major of Life Science, Division of Bioconvergence, College of Convergence and Integrated Science, Kyonggi University, 154-42 Gwanggosan-ro, Yeongtong-gu, Suwon-si, Gyeonggi-do 16227, Korea; sslee@kyonggi.ac.kr

**Keywords:** 5-lipoxygenase inhibitor, zileuton, BK channel, leukotriene B4, angiogenesis, Erg

## Abstract

The arachidonic acid metabolism through 5-lipoxygenase (5-LO) pathways is involved in modulating both tumorigenesis and angiogenesis. Although anti-carcinogenic activities of certain 5-LO inhibitors have been reported, the role of zileuton, a well known 5-LO inhibitor, on the endothelial cell proliferation and angiogenesis has not been fully elucidated. Here, we report that zileuton has an anti-angiogenic effect, and the underlying mechanisms involved activation of the large-conductance Ca^2+^-activated K^+^ (BK) channel. Our results show that zileuton significantly prevented vascular endothelial growth factor (VEGF)-induced proliferation of human umbilical vein endothelial cells (HUVECs) in vitro, as well as in vivo. However, such anti-angiogenic effect of zileuton was abolished by iberiotoxin (IBTX), a BK channel blocker, suggesting zileuton-induced activation of BK channel was critical for the observed anti-angiogenic effect of zileuton. Furthermore, the anti-angiogenic effect of zileuton was, at least, due to the activation of pro-apoptotic signaling cascades which was also abolished by IBTX. Additionally, zileuton suppressed the expression of VCAM-1, ICAM-1, ETS related gene (Erg) and the production of nitric oxide (NO). Taken together, our results show that zileuton prevents angiogenesis by activating the BK channel dependent-apoptotic pathway, thus highlighting its therapeutic capacity in angiogenesis-related diseases, such as cancer.

## 1. Introduction

Angiogenesis, growth of new blood vessels by endothelial cells (ECs), is essential for normal development, as well as pathologic conditions, including tumor development, thrombosis, rheumatoid arthritis, and diabetic retinopathy [1,2,3]. The interaction between tumor cells and vascular ECs induces various growth factors, the expression of cytokines, chemokines, and others [4,5]. Among these known angiogenic molecules, vascular endothelial growth factor (VEGF) is the most important factor promoting angiogenesis [6,7]. Angiogenesis involves degradation of the extracellular matrix (ECM) of the parental vessels, which induce the migration, proliferation, and the formation of the tube-like structure of EC. Thus, blockade of angiogenesis by inhibiting angiogenic factors could be an attractive option to prevent tumor growth.

Found in many cells, including endothelial cells, vascular smooth muscle cells, and cancer cells, large-conductance Ca^2+^-activated K^+^ channel (BK channel) may contribute to cell proliferation and migration [8,9]. Dysregulation or upregulation of BK channels have been associated with altered cell proliferation and migration [10,11,12], which are key features of cancer development and progression [13,14]. In this regard, activation of BK channel may play a key role in developing tumors of breast, prostate, and glioma [15,16]. A previous study reported that BK channel antagonist inhibits proliferation of breast cancer cells [17]. This showed that BK channel antagonist exerts anti-proliferative and anti-invasive activities against breast cancer through the reduction of BK channel expression, secretion of TNF-α and upregulation of G1 cell cycle arrest protein p*27*. In contrast, BK channel activation by NS1619, a BK channel activator, attenuated proliferation of ovarian cancer cells [18]. An increase of p53 and its downstream target, p*21*^Cip1^, and Bax by NS1619 are responsible for the apoptotic effect induced by opening of BK. Besides its effect in the cancer cells, BK channel in endothelium has been shown to regulate the vascular functions and thereby influences vascular remodeling and angiogenesis. There are reports that the activation of BK channel plays a necessary role in mediating the anti-proliferative and anti-angiogenic effects in human umbilical vein endothelial cells (HUVECs) [19]. Specifically, BK channels modulate membrane action potential, cell death in neurons, and apoptosis in endothelial cells [19,20].

It has been suggested that phospholipase A_2_ (PLA_2_)/arachidonic acid (AA) pathways are associated with BK channel activity. For example, for the relaxation of coronary artery smooth muscle cells, AA release by H_2_O_2_ stimulation regulates BK channel activity, most likely via lipoxygenase activity [21]. 5-Lipoxygenase (5-LO) contributes to the synthesis of leukotriene eicosanoids from arachidonic acid (AA) [22,23]. Leukotriens, downstream metabolites of 5-LO pathway, trigger various responses such as smooth muscle contraction, leukocyte chemotaxis, and elevated vascular permeability. The 5-LO signaling plays an important role in the progression of asthma [24] and inflammatory diseases [23] as well. Some reports suggested that the 5-LO pathway may also be associated with tumorigenesis, particularly in prostatic cancer and certain forms of leukemia [25]. Moreover, leukotriene B4 (LTB4) also seems to modulate colon cancer growth via interaction with its receptor, BLT1 [26]. These findings imply that 5-LO inhibitors may serve as a viable option for cancer treatment. To date, the potential of 5-LO inhibitors as anti-cancer therapeutics has been continuously scrutinized. For example, it was reported that 5-LO inhibitors can control the process of mammary tumor development [27]. Nevertheless, functions of BK channel activity and lipoxygenase metabolism in angiogenesis are largely unknown. Additionally, it is well known that EC apoptosis plays an important role in the vascular regression, remodeling, and angiogenesis under physiologic or pathological conditions [28,29,30]. Indeed, several in vivo studies have demonstrated that EC apoptosis could be an effective modality against cancer.

Therefore, the aim of the current study was to examine the effect of 5-LO inhibitor zileuton, which has been approved for clinical use for the treatment of asthma, on VEGF-induced angiogenesis focusing on BK channels as potent mediators.

## 2. Materials and Methods

### 2.1. Materials

Zileuton was purchased from Tocris Bioscience (Ellisville, MO, USA). NS1619 and iberiotoxin (IBTX) were obtained from Sigma (St. Louis, MO, USA). Recombinant human VEGF was acquired from R&D Systems (Minneapolis, MN, USA). BrdU assay kit (colorimetric), LTB4 kits, and both of cysteinyl leukotrienes (Cys-LTs) kits and caspACE™ assay kit were obtained from Roche applied science (Penzberg, Germany), Enzo Life Sciences (Seoul, Korea), and Abcam (Cambridge, MA, USA), respectively. PGE_2_ and 6-keto PGF_1__α_ (the stable hydrolysis products of PGI_2_) were from Enzo Life Science (Seoul, Korea) and Cayman chemicals (Ann Arbor, MI, USA). Specific antibodies against ICAM-1, VCAM-1, eNOS^Ser1179^ and Erg1/2/3, Bcl-2 and Bax were obtained from Santa Cruz Biotechnology (Santa Cruz, CA, USA). All other reagents were purchased from Sigma (St. Louis, MO, USA) unless otherwise noted.

### 2.2. Animal and Ethics Statement

Male C57BL6/J (8–9 wks) mice, weighing 20–25 g, were purchased from Dae-Han Experimental Animal Center (Dae-Han Biolink, Eumsung, Republic of Korea) and maintained under specific pathogen-free (SPF) conditions at animal breeding facility of National Institute of Health in Korea (KNIH). All animal experiments were performed in accordance with the guidelines for the care and use of laboratory animals by the KNIH. The protocols were reviewed and approved by the Animal Ethics Committee of KNIH (confirmation number: KCDC-038-13). All surgery was performed under sodium pentobarbital anesthesia, and all efforts were made to minimize suffering.

### 2.3. Cell Cultures

HUVECs (endothelial cell line derived from human umbilical cord vein) were purchased from ATCC (CRL-1730) and cultured using Endothelial Cell Growth Medium-2 (EGM-2; Clonetics, San Diego, CA, USA) supplemented with 10% heat-inactivated fetal bovine serum and 1% penicillin–streptomycin mixture (Invitrogen, Carlsbad, CA, USA) at 37 °C with 5% CO_2_. Cells were grown to confluence on 0.1% gelatin-coated culture dishes and used for experiments within passage 8 to avoid senescence. To induce growth arrest, sub-confluent HUVECs were maintained by low serum medium (EBM-2 containing 0.1% FBS) for 4 h and used for experiments.

### 2.4. Cell Viability Assay

HUVECs seeded at a density of 1 × 10^5^ cells/well in 12 well plates were incubated with various concentration of zileuton (1–50 µM) for 1 h and then exposed to VEGF (10 ng/mL) for 24 h. At the end of the treatment, MTT assay was carried out as previously reported [31]. Briefly, cells were cultured with MTT at a final concentration of 0.5 mg/mL for 4 h. Produced purple formazan crystals were dissolved using DMSO and the optical density (OD) was measured at 570 nm using a Perkin Elmer VictorX4 (Waltham, MA, USA) microplate reader.

### 2.5. BrdU Incorporation Assay

BrdU incorporation assay was performed using Cell Proliferation ELISA, BrdU colorimetric Kit. Briefly, cells were seeded at 5 × 10^3^/100 µL/well in 96 well plates and low serum medium (0.1% FBS) were used for starvation. After exposure of zileuton for 1 h, cells were stimulated with VEGF (10 ng/mL) for additional 24 h. Then, the cells were labeled using 10 µM BrdU per well and re-reincubated for further 4 h at 37 °C in a humidified atmosphere. After incubation, the culture media was removed and the cells were fixed by adding FixDenat. Next, the cells were incubated with the anti-BrdU-peroxidase (POD) antibody for 90 min at room temperature. After the removal of excess anti-BrdU-POD antibody, the cells were washed and the substrate solution was added. The reaction product was quantified by measuring the absorbance using a multi-well spectrophotometer at 450 nm.

### 2.6. Scratch Wound Healing Assays

To evaluate the effect of zileuton in cell migration, wound healing assay was carried out using 35 mm µ-dish with culture insert (IBID GmbH, Martinsried, Germany). Briefly, HUVECs (1 × 10^5^ cells/well) were seeded into reservoir of an IBIDI insert. When cells’ confluence reached over 80%, the insert was removed by creating a gap of ~500 µm, and cells were treated with various concentrations of zileuton (1–50 µM) 1 h before and then exposed to VEGF (10 ng/mL) for 24 h with FBS free medium. The closure of the gap was observed and photographs of three fields of view were taken at 24 h using inverted microscope (OLYMPUS CK40, Japan). The migration distance was quantified using Vimasis software (IBID GmbH, Martinsried, Germany)

### 2.7. Tube Formation Assay

To identify the antiangiogenic effect of zileuton, tube formation assay was conducted on Matrigel (BD Biosciences, Bedford, MA, USA)-coated plates as previously reported [32]. Firstly, the growth factor-reduced Matrigel was melted at 4 °C and then plated 300 µL/well Matrigel into 24-well plates. The Matrigel-coated plate was placed on ice at a horizontal level for 10 min to distribute Matrigel evenly and then incubated at 37 °C for 30 min polymerization. Then, cells (1 × 10^5^ cells/well) were seeded on the Matrigel-coated plate and pretreated with or without IBTX (0.3 µM, BK channel blocker) for 30 min. After pretreatment of IBTX, cells were treated with either zileuton (1–50 µM) or NS1619 (10 µM) for 1 h and then followed by the addition of VEGF (10 ng/mL) for indicated time points (4–6 h after VEGF stimulation) with 5% CO_2_ at 37 °C. Images and quantification of endothelial network formation was performed by counting the number of tubes formed per field in each well using an inverted (bright-field) microscope (Nikon) to evaluate the antiangiogenic capacity of zileuton.

### 2.8. Matrigel Plug Assay and Analysis of Hemoglobin Content

The effects of zileuton on angiogenesis in vivo were monitored using Matrigel plug assay by using growth factor-reduced Matrigel (in liquid at 4 °C). Prepared Matrigel (0.5 mL) containing 40 units/mL heparin and 10 ng/mL VEGF in the presence or absence of zileuton (50 µM) was injected into the anesthetized mice subcutaneously around the flanks area. Animals were randomly divided into 3 groups and administered the following: (a) PBS (used as a control); (b) VEGF (10 ng/mL) alone; (c) co-treatment of VEGF (10 ng/mL) and zileuton (50 µM). Mice were euthanized by CO_2_ at day 7 for further analysis. Removed Matrigel plugs were fixed in 10% neutral-buffered formalin, processed for embedding in paraffin, sectioned, and stained with hematoxylin and eosin (H&E). To quantitate the vascularization of the plug, the amount of hemoglobin (Hb) accumulated in the plug was measured using total Hb kits following the manufacturer’s protocol. Briefly, harvested plugs were weighted and incubated overnight in de-ionized water at 37 °C. Then, plugs were homogenized and centrifuged at 7000*g* for 15 min at 4 °C. Hb content was quantified by directly measuring the supernatants at OD405, calculated against as standard curve generated with purified porcine hemoglobin (Sigma-Aldrich, St. Louis, MO, USA), and normalized against wet weight of the plug.

### 2.9. Measurement of Nitric Oxide (NO) Production

The cell culture media were collected and assayed for NO production using a commercial kit according to the manufacturer’s protocol for Griess Reagent System (Promega, Seoul, Korea). Briefly, 50 μL of each sample was transferred to a 96 multi-well plate. Each sample was then incubated with 50 μL of Sulfanilamide Solution for 10 min, followed by a second incubation with 50 μl of N-1-napthylethylenediamine dihydrochloride (NED) Solution for 10 min. Absorbance was measured at 540 nm by using microplate reader and NO levels were calculated using a nitrite standard reference curve. Then, they were expressed as the % of control.

### 2.10. BK Channel Small Interfering RNA (siRNA) Transfection

For BK channel siRNA transfection, HUVECs were transfected with either BK channel targeted siRNA (30 nM; Santa Cruz Biotechnology) or control siRNA using Lipofectamine^TM^ RNAiMAX (Invitrogen, Carlsbad, CA, USA) in Opti-MEM medium according to the manufacturer’s instructions. Cells were incubated for 12 h, media were replaced with normal culture medium. After 24 h incubation, cells were exposed to VEGF (10 ng/mL) for 6 h (for the tube formation assay) or 24 h (for the migration or BrdU assay) in the absence or presence of zileuton (50 µM). Efficiency of BK channel knockdown was measured by Western blot analysis against BK channel antibody.

### 2.11. Assay of LTB_4_, PGE_2_, PGI_2_, and Caspase-3 Activity

The levels of LTB_4_, PGE_2_, 6-keto PGF_1__α_ (PGI_2_), and activity of caspase-3 were measured by using commercial ELISA kits according to manufacturer’s protocol and detailed protocol was mentioned in previous report [31,32]. To determine the LTB_4_ assay and caspase-3 activity, cells were treated with either zileuton (50 μM) and NS1619 (10 µM) for 1 h before exposure to VEGF (10 ng/mL) for 24 h in the presence or absence of IBTX (0.3 µM). To determine the PGE_2_ and PGI_2_, cells were treated with either zileuton (50 μM) and indomethcin (10 µM) for 1 h before exposure to VEGF (10 ng/mL) for 15 or 24 h. Following treatment, supernatant (100 μl) were incubated with polyclonal LTB_4_, PGE_2_, and PGI_2_ binding wells for recommended time periods. After stopping of enzymatic reaction, optical density was measured at 405 nm. For the caspase-3 activity, the cells were collected and resuspended in lysis buffer containing 50 mmol/L HEPES, pH 7.4, 0.1% CHAPS, 1 mmol/L DTT, 0.1 mmol/L EDTA, and 0.1% Triton X-100. Following incubation for 30 min on ice, cell lysate was centrifuged at 11,000*g* for 10 min at 4 °C, and the protein concentration in the supernatants was measured using the Bradford dye method. The supernatants were incubated with reaction buffer containing 2 mmol/L Ac-DEVD-AFC, a fluorogenic substrate for caspase-3 in a caspase assay buffer at 37 °C with 10 mmol/L DTT for 30 min. Caspase-3 activity was determined by measuring the absorbance (405 nm).

### 2.12. RNA Preparation and Real-Time PCR

Total RNA was extracted from cells using easy-BLUE Total RNA extraction kit (Intron Biotechnology). RNA (500 ng) was reverse transcribed to cDNA using TOPscriptTM RT Dry MIX (Enzynomics, Daejeon, Korea). Human PCR primers against ICAM-1, VCAM-1, and GAPDH were as follows: ICAM-1, Sense-5′-ATGCCCAGACATCTGTGTCC-3′, Antisense-5′-GGGGTCTCTATGCCCAACAA-3′; VCAM-1, Sense-5′- GGGAAGATGGTCGTGATCCTT-3′, Antisense-5′- TCTGGGGTGGTCTCGATTTTA-3′; GAPDH, Sense-5′- CTGGGCTACACTGAGCACC -3′, Antisense-5′- AAGTGGTCGTTGAGGGCAATG -3′. Real-time PCR was performed in triplicate using a SYBR Green Master mix (Toyobo, Japan) using Light cycler 1.5 (TAKARA, Seoul, Korea). The expression levels of the target genes were calculated versus GAPDH.

### 2.13. Western Blot Analysis

Cells were lysed with 1× protein lysis buffer (Cell Signaling Technology, Danvers, MA, USA) and centrifuged at 16,000 *g* for 20 min. Aliquots of lysates were subjected to measure protein contents using Bio-rad protein assay reagent, equal amounts of protein were loaded into 10% SDS gels and transferred to PVDF membranes. Blots were added with appropriate primary antibodies including ICAM-1, VCAM-1, Bcl-2, Bax, BK channel, and β-actin, and then incubated overnight at 4 °C. After washing with Tris-buffered saline/Tween 20 (TBS-T), secondary antibody conjugated with horseradish peroxidase (HRP) was added in the blots. Immunoreactive bands were detected using chemiluminescence kits (ECL; Amersham Biosciences, Little Chalfont, Buckinghamshire, UK). The relative band densities were determined by Image J software.

### 2.14. Whole-Cell Patch Clamp Recordings

The whole-cell configuration of the patch-clamp technique was performed at the RUDACURE corporation (http://www.redacure.com; Inchon, Korea) with HEKA EPC10 amplifier (HEKA instrument, Germany), and the patch pipettes were pulled from borosilicate capillaries (Chase Scientific Glass Inc., Rockwood, CA, USA). When filled with the pipette solution, the resistance of the pipettes was 4–5 MΩ. The recording chamber (volume 300 uL) was continuously superfused (2–3 mL/min). Series resistance was compensated for (>80%) and leak subtraction was performed. Data were low-pass filtered at 2 kHz and sampled at 10 kHz. Patchmaster (HEKA Instruments) software was used for experiments and analysis. The pipette solution for voltage-clamp experiments contained (in mM) 125 K-gluconate, 5 KCl, 5 NaCl, 2.5 CaCl_2,_ 2 MgCl_2,_ 5 EGTA, and 5 HEPES, adjusted to pH 7.3 with KOH. The extracellular solution for voltage-clamp experiments contained (in mM) 140 NaCl, 5 KCl, 1 MgCl_2_, 10 HEPES, 10 glucose, and 2 EGTA, adjusted to pH 7.3 with NaOH. Voltage-clamp experiments were performed at a holding potential of −60 mV.

### 2.15. Measurement of Intracellular Ca^2+^

Intracellular Ca^2+^ was determined using fluorescent Ca^2+^ indicator fura-2-acetoxymethyl ester (AM) (Molecular probes, Seoul, Korea). HUVEC cells were seeded at 5 × 10^4^/ well in a 0.1% gelatin-coated black 96-well plate (Corning, NY, USA) overnight. Then, cells were washed with Krebs-Ringer HEPES (KRH) buffer, including 138 mM NaCl_2_, 5 mM KCl, 1.3 mM CaCl_2_, 1.3 mM MgCl_2_, 24 mM NaHCO_3_, 2 mM KH_2_PO_4_, 10 mM Na_2_HPO_4_, 25 mM HEPES, and 10 mM glucose. After washing with KRH buffer, cells were incubated with fura-2 AM (4 µM) for 30 min in KRH buffer without CaCl_2_, then washed, left for 30 min to allow complete de-esterification of fura-2 AM in complete KRH solution. For drug effects, zileuton (50 µM) or NS1619 (10 µM) in the presence or absence of VEGF (10 ng/mL) were added then, intracellular Ca^2+^ influx was measured. Intracellular Ca^2+^ rise was monitored by measuring the ratio of 510 nm emission using the Nikon TS 100 fluorescence imaging system (Nikon Instrument Inc., USA) according to the manufacturer’s guideline and changes in intracellular Ca^2+^ fluctuation as function of time were plotted and analyzed by the InCyt Im2 software (University of Cincinnati, USA).

### 2.16. Statistical Analysis

The experimental results were expressed as mean ± SD values, and statistically significant differences were determined using Student’s *t*-tests for comparisons between two experimental groups and one-way ANOVA followed by Tukey’s posthoc test for comparisons among multiple groups. A p-value of less than 0.05 was considered statistically significant. Path clamp results were expressed as mean ± SEM. * *p* < 0.05 and ** *p* < 0.01 versus the control group as determined by two-way ANOVA with Fisher’s LSD test. Statistical analysis was performed using the SPSS18.0 software (SPSS Inc., Chicago. IL).

## 3. Results

### 3.1. Zileuton Inhibits VEGF-Induced Angiogenesis In Vitro

**Figure 1:**
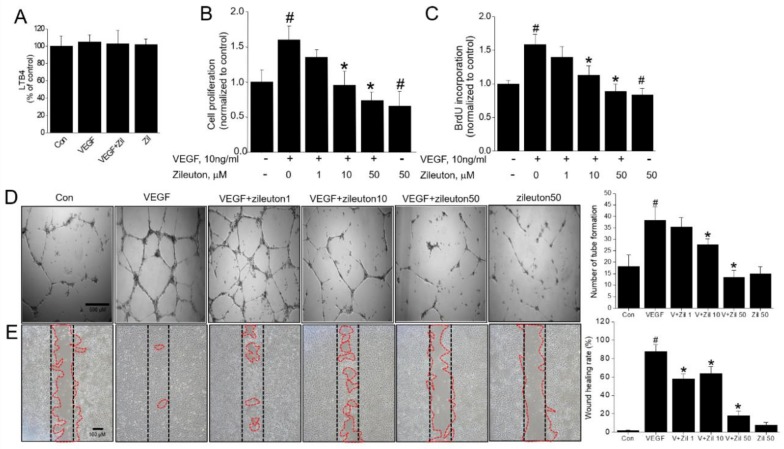
Effects of zileuton on vascular endothelial growth factor (VEGF)-induced proliferation and migration of HUVECs. Human umbilical vein endothelial cells (HUVECs) were incubated with varying concentrations of zileuton (1, 10, 50 µM) for 1 h and then stimulated with VEGF (10 ng/mL) for 24 h. (**A**) Leukotriene B4 (LTB_4_) production was determined by LTB_4_ Elisa kits. (**B**) Cell proliferation was assessed by MTT assay and (**C**) DNA synthesis was assessed by the BrdU incorporation assay. (**D**) Representative photomicrographs of the tube formation assay results. HUVECs were pretreated with or without zileuton (1, 10, 50 µM) for 1 h prior to seeding the cells on Matrigel. Trypsinized cells were plated onto the surface of growth factor-induced Matrigel (8 × 10^4^ cells/well) and treated with VEGF (10 ng/mL) for 6 h, and tube formation was observed using microscopy. (**E**) Wound healing and migration assays are done by seeding cells into the Culture-Insert. Cells were pretreated with zileuton for 1 h before the induction of cellular migration by VEGF (10 ng/mL). After cell attachment, a cell-free gap is created in which the cell migration can be visualized and a photograph was taken after 24 h of incubation. Gap closure was measured for quantification (red line) using Image J program. Data shown represent the means ± SD from three independent experiments (each performed in duplicate). ^#^ *p* < 0.05 versus non-treated cells, * *p* < 0.05 versus VEGF-treated cells.

We firstly wanted to determine whether VEGF could induce the two known mediators of 5-LO action, LTB_4_ and cysteinyl leukotrienes (Cys-LTs). The production of LTB4 and Cys-LTs (LTC_4_, LTD_4_, and LTE_4_) during VEGF-induced angiogenesis was assessed. In our data, both of LTB_4_ (Figure 1A) and Cys-LTs (data not shown) were not increased by VEGF treatment, indicating the 5-LO-LTB_4_ or Cys-LTs pathway is not activated in VEGF-stimulated HUVECs (Figure 1A). We next investigated whether the COX-2 pathway is involved in the VEGF-induced angiogenesis. Stimulation of VEGF for 15 or 24 h in HUVECs resulted in increased 6-keto PGF1_α_ (the stable hydrolysis products of PGI_2_), indicating that COX-2 pathway is requisite step in angiogenesis. In addition, pretreatment with either zileuton (50 µM) or indomethacin (10 µM) completely abolished PGI_2_ following treatment with VEGF, suggesting that zileuton’s action in HUVECs was simply related to the inhibition of prostanoid such as PGI_2_ (Figure S1). In contrast, PGE_2_ was unresponsive to VEGF treatment (data not shown). As shown in Figure 1B, 24 h of VEGF treatment significantly increased the growth of HUVECs, as analyzed by the MTT assay. However, this was attenuated by zileuton in a concentration-dependent manner (Figure 1B). The results of BrdU incorporation labeling assay indicated that zileuton inhibited DNA synthesis as well (Figure 1C). In tube formation assay, zileuton concentration-dependently prevented the VEGF-induced tube formation, with maximal inhibition at 50 µM (Figure 1D). HUVEC migration was assessed by wound scratch closure assay, and as shown in Figure 1E, zileuton significantly prevented cell migration in a concentration dependent manner.

### 3.2. Zileuton Inhibits Angiogenesis In Vivo

**Figure 2:**
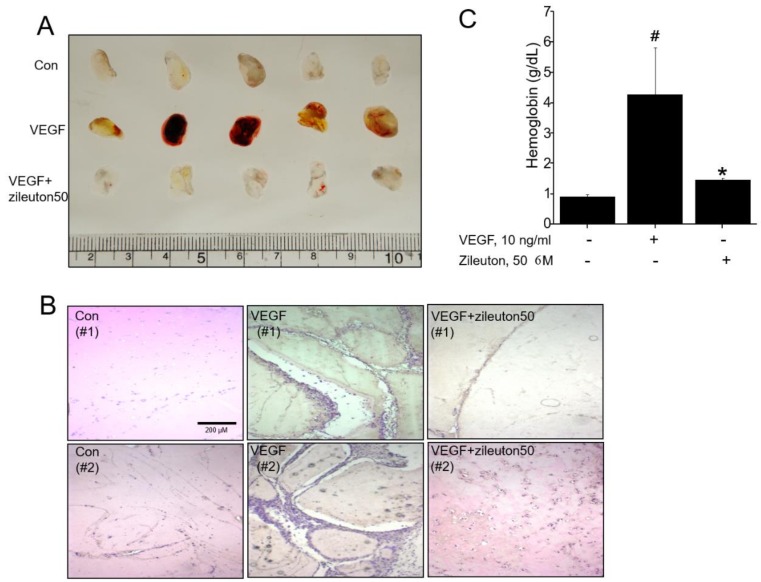
Zileuton inhibits VEGF-induced angiogenesis in a mouse Matrigel model. Mice (*n* = 5) were injected subcutaneously with or without zileuton (50 µM) containing Matrigel with VEGF (100 ng/mL). (**A**) Plugs were excised from the mice after 1 week and photographed. (**B**) Histological analysis was performed using H&E staining following excision of the Matrigel plug. (**C**) Quantification of the hemoglobin (Hb) content of Matrigel plugs by spectrophotometry at 540 nm. Data shown represent the means ± SD from three independent experiments. ^#^ *p* < 0.05 versus non-treated group, * *p* < 0.05 versus VEGF-treated group.

The effect of zileuton angiogenesis in vivo was examined by using Matrigel plug assay. The VEGF containing Matrigel plugs were subcutaneously implanted in mice with or without zileuton for one week. Zileuton (50 μM) significantly inhibited VEGF-induced angiogenesis as shown in Figure 2A. Blood vessel formation was induced in Matrigel containing VEGF (10 ng/mL), but this was suppressed by zileuton (Figure 2B). The hemoglobin (Hb) content in plugs, which was used as a parameter to quantify angiogenesis, increased in the VEGF-containing plugs compared to the normal controls. However, again, zileuton significantly decreased the Hb content by 94%, compared to the VEGF-treated controls (Figure 2C). These results strongly suggest the antiangiogenic efficacy of zileuton in vivo.

### 3.3. Zileuton Inhibits VEGF-Induced Adhesion Molecules, Erg, and Production of NO

**Figure 3:**
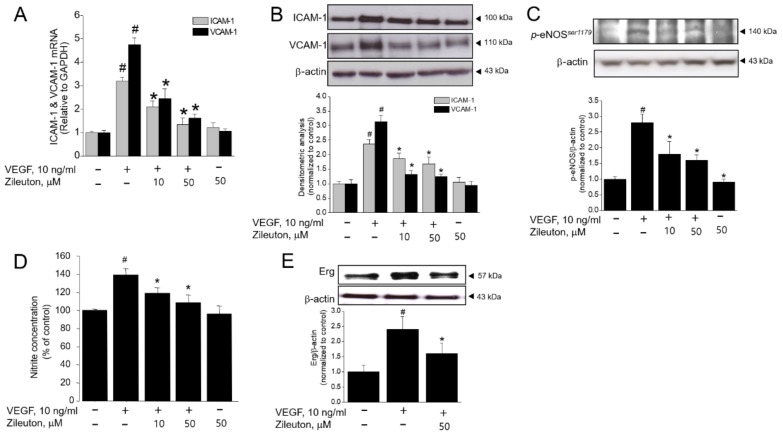
Effects of zileuton on VEGF-induced angiogenic factors in HUVECs. Cells were pretreated with zileuton (10 and 50 µM) for 1 h prior to VEGF treatment (10 ng/mL). After 8 h (qPCR) or 24 h (Western blot, Nitrite levels) stimulation with VEGF, (**A**) qPCR was performed for ICAM-1 and VCAM-1. (**B–C**, **E**) Western blot analysis was performed using antibodies against ICAM-1, VCAM-1, eNOS^ser1179^, and Erg. (**D**) Nitrite levels were measured by Griess reagent kit. Protein expressions were quantified by densitometry and normalized by their control. Immunoblots shown are representative of three independent experiments. ^#^ *p* < 0.05 versus non-treated cells and * *p* < 0.05 versus VEGF-treated cells.

Cell adhesion molecules are also critical for angiogenesis, endothelial migration, proliferation, and differentiation. Therefore, the effect of zileuton on the VEGF-induced expression of ICAM-1 and VCAM-1 was examined. As shown in Figure 3A,B, VEGF increased ICAM-1, VCAM-1 mRNA, and protein expression, which were all decreased significantly with zileuton treatment in a concentration dependent manner. Since NO is a mediator of pro-angiogenic factors [33,34], the influence of NO against the zileuton-induced antiangiogenic effects was further examined. VEGF induced the production of NO and phosphorylation of eNOS^ser1179^, whereas zileuton decreased eNOSser^1179^ phosphorylation and NO production (Figure 3C,D). Additionally, VEGF significantly increased Erg expression, which modulates the transcription of key adhesion molecules such as ICAM-1 and VCAM-1. However, this was also abolished by zileuton (Figure 3E).

### 3.4. Zileuton Augments Whole-Cell K^+^ Outward Currents in HUVECs

**Figure 4:**
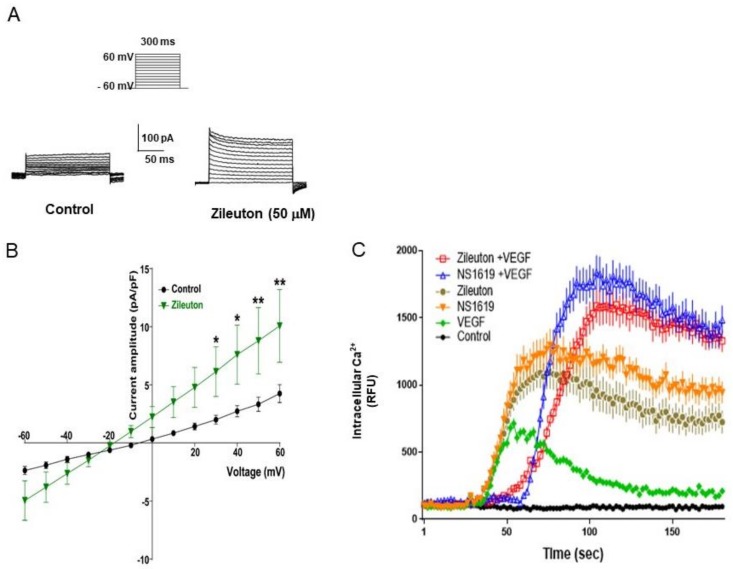
Effects of zileuton on the amplitude of whole-cell current in HUVECs. (**A**) Sample traces of whole-cell currents to control (*n* = 9) and zileuton (50 μM, *n* = 12) produced by stepwise depolarization, in steps of 10 mV, from −60 to +60 mV with a holding potential of −60 mV. (**B**) Current–voltage relationship for steady-state current (holding potential = −60 mV) under control conditions. Values are the mean ± SEM. * *p* < 0.05 and ** *p* < 0.01 versus the control group. The significance was determined by two-way ANOVA with Fisher’s LSD test. (**C**) Typical traces showing changes in intracellular Ca^2+^ in HUVECs after addition of zileuton (50 µM), NS1619 (10 µM) with or without VEGF (10 ng/mL). Data shown represent the means ± SD from three independent experiments. * *p* < 0.05 and ** *p* < 0.01 versus non-treated control cells, respectively.

Using whole-cell patch clamp recording, the effect of zileuton on K^+^ current regulation in HUVECs was examined. As shown in Figure 4A, the K^+^ outward currents were augmented in the presence of zileuton (50 µM) compared to control. In addition, the currents–voltage relationship for steady-state current (holding potential = −60 mV) is shown in Figure 4B. On a holding potential of −60 mV, step depolarization from −60 mV to +60 mV was measured to elicit a voltage-dependent outward current. The results indicated that zileuton showed a tendency of increasing outward K+ currents (Figure 4A,B). Since intracellular Ca^2+^ is accumulated by BK channel opening, the effect of zileuton on the intracellular Ca^2+^ level was further examined. According to the data, although zileuton, NS1619, and VEGF increased the intracellular Ca^2+^, the magnitude was smallest in the VEGF treated group. Additionally, when cells were treated with zileuton or NS1619 in the presence of VEGF, the intracellular Ca^2+^ synergistically increased with a bit delayed peak (Figure 4C).

### 3.5. Zileuton Attenuates VEGF-Induced Angiogenesis and Proliferation via BK Channel Opening

**Figure 5:**
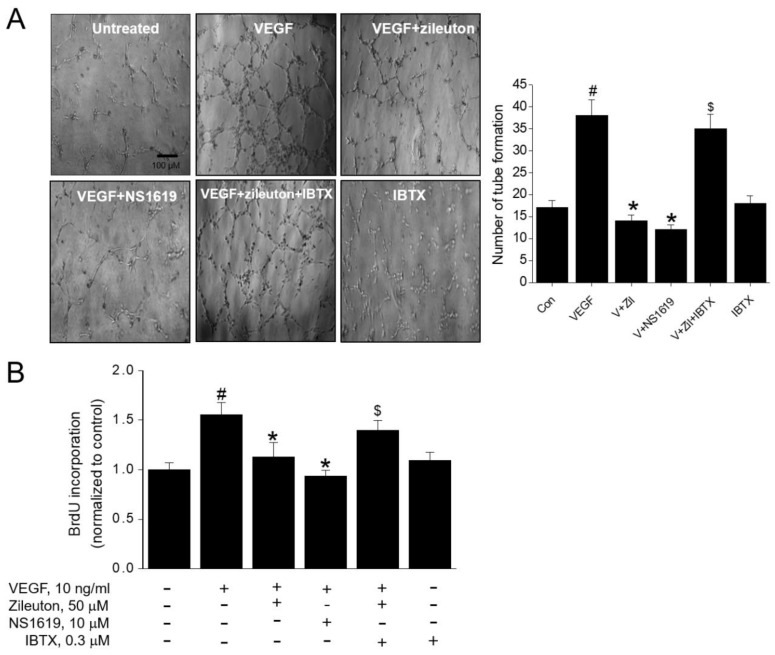
Effects of zileuton on VEGF-induced angiogenesis and proliferation via large-conductance Ca^2+^-activated K^+^ (BK) channel opening on HUVECs. Cells were pre-incubated with zileuton (50 µM) or NS1619 (10 µM) for 1 h in the absence or presence of 0.5 µM iberiotoxin (IBTX) and then treated with VEGF (10 ng/mL). (**A**) After 4 h stimulation with VEGF, tube formation was observed under a microscope. (**B**) After 24 h stimulation with VEGF, DNA synthesis was assessed using the BrdU incorporation assay. Data shown represent the means ± SD from four independent experiments (each performed in duplicate). ^#^ *p* < 0.05 versus non-treated cells, * *p* < 0.05 versus VEGF-treated cells, ^$^ *p* < 0.05 versus the VEGF plus zileuton-treated cells.

BK channels are involved in VEGF-induced angiogenesis [19]. Involvement of BK channel was examined using capillary tube formation and BrdU incorporation assays. As shown in Figure 5A, zileuton (50 µM) suppressed VEGF-induced tube formation, which was reversed by IBTX, a BK channel blocker. Furthermore, a BK channel opener NS1619 (10 µM) also inhibited VEGF-induced tube formation. These data strongly suggested that the observed anti-angiogenic effect of zileuton is mediated by the BK channel opening. In BrdU incorporation assay, IBTX abrogated the anti-proliferative action of zileuton and NS-1619 also inhibited VEGF-induced cell proliferation (Figure 5B).

### 3.6. Effect of BK Channel Knockdown on Zileuton-Induced Antiangiogenic and Anti-Proliferative Activity

**Figure 6:**
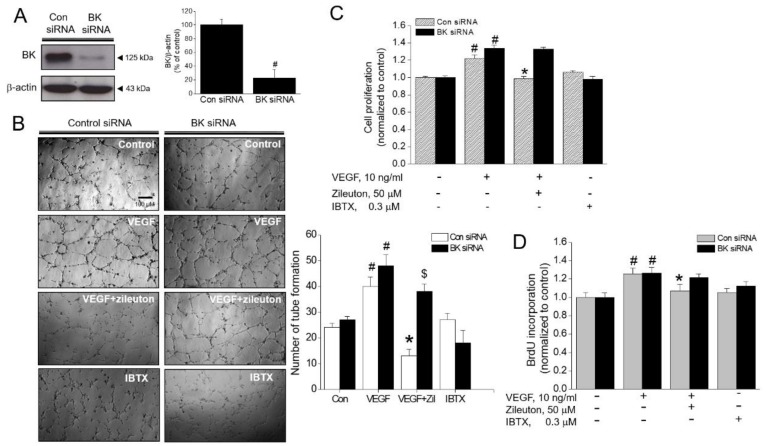
BK channel silencing abolishes zileuton-induced antiangiogenic effects in VEGF-treated HUVECs. Cells were transfected with a BK channel-specific siRNA (30 nM) for 36 h and pre-incubated with zileuton (50 µM) for 1 h then treated with VEGF (10 ng/mL). (**A**) Total protein was isolated and analyzed by immunoblotting using antibodies against BK channel and β-actin. (**B**) After 6 h stimulation with VEGF, tube formation was observed under a microscope. After 24 h stimulation with VEGF, (**C**) cell proliferation was measured by MTT assay, (**D**) DNA synthesis was assessed using the BrdU incorporation assay. Data shown represent the means ± SD from three independent experiments (each performed in duplicate). ^#^ *p* < 0.05 versus untreated control, * *p* < 0.05 versus the VEGF-treated cells. ^$^ *p* < 0.05 versus the VEGF plus zileuton-treated cells.

To further evaluate the involvement of BK channel, siRNA specific to BK channel was utilized. The siRNA (30 nM) decreased the expression of BK channel up to 80% (Figure 6A). With these BK channel knock-downed HUVECs, zileuton was ineffective in suppressing VEGF-induced tubular network formation and cell proliferation (Figure 6B–D), strongly implying that BK channel is required for the action of zileuton to exert the anti-angiogenic and anti-proliferative effects.

### 3.7. Zileuton Induces Apoptosis Through BK Channel in VEGF-Induced Angiogenenic HUVECs

**Figure 7:**
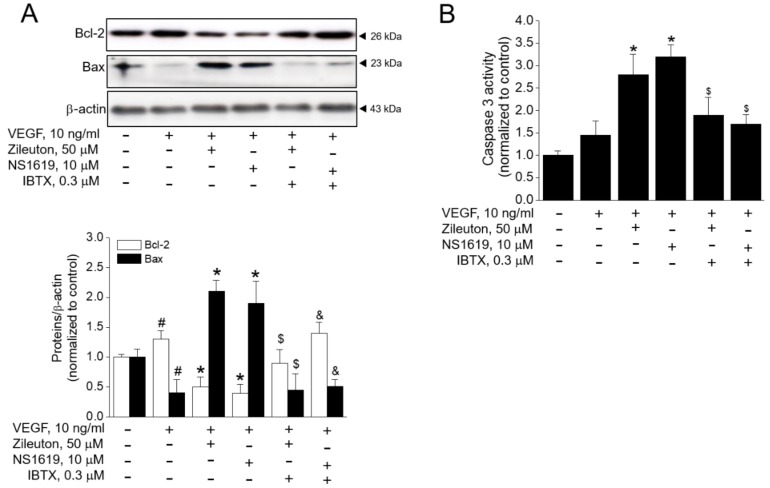
Effect of zileuton on the regulation of Bcl-2, Bax, and caspase-3 activity in HUVECs. Cells were pre-treated with zileuton (50 µM) or NS1619 for 1 h in the presence or absence of IBTX, then treated with VEGF (10 ng/mL) for 24 h. (**A**) Total protein was isolated and analyzed by immunoblotting using antibodies against Bcl-2, Bax, and β-actin. (**B**) Caspase-3 activity was measured by colorimetric assay kit. Bcl-2 and Bax expressions were quantified by densitometry and normalized by β-actin. Data shown represent the means ± SD from three independent experiments. ^#^ *p* < 0.05 versus untreated control, * *p* < 0.05 versus the VEGF-treated cell, ^$^ *p* < 0.05 versus the VEGF plus zileuton-treated cells, ^&^ *p* < 0.05 versus the VEGF plus NS1619-treated cells.

To more closely examine whether antiangiogenic and antiproliferative action of zileuton are related with apoptosis, we checked Bcl-2 expression as an anti-apoptotic, Bax expression and caspase-3 activity as a proapoptotic. VEGF increased the expression of Bcl-2, but this was abrogated by zileuton. On the other hand, the proapoptotic Bax expression was decreased by VEGF, while zileuton increased the expression of Bax (Figure 7A) and caspase-3 activity (Figure 7B). In addition, NS1619 had an effect similar to zileuton on the expression of Bcl-2, Bax, and the activity of caspase-3. Since such apoptotic effects of zileuton or NS1619 in VEGF-induced angiogenesis were abrogated with IBTX, it was speculated that the effect of zileuton involved BK channel opening.

## 4. Discussion

Angiogenesis has been described as a hallmark of tumor progression, and extensive evidence suggest that anti-angiogenic strategy may be an attractive modality for anticancer therapy [3]. Thus, interests have been focused on the development or identification of anti-angiogenic agents. Additionally, several studies have shown that eicosanoids produced by 5-LO play important roles in cancer development [25]. Inhibition of cancer cell growth and apoptotic induction by 5-LO inhibitors have been reported in various cancers [35,36,37]. Despite a long-lasting effort to develop 5-LO inhibitors, zileuton is the only 5-LO inhibitor on the market for the treatment of asthma [38]. Evidence indicates that zileuton significantly inhibits cardiomyocytes apoptosis [39], ER stress in myotubes [40], chemically-induced lung cancer in mice [41], and pancreatic cancer cells [42]. However, the clinical usefulness of zileuton in cancer development and angiogenesis, as well as its precise mechanism of action, remains to be clarified. In the present study, we explored the potential use of zileuton as an anti-angiogenic drug. Major finding of the present study is that the anti-angiogenic action of zileuton is, at least in part, contributed to the BK channel-dependent apoptosis in HUVECs.

In the last few decades, there has been a significant increase in the number of reports examining the 5-LO pathway and its metabolites with hopes of controlling cancer development and progression. Many lines of experimental evidence suggest that the arachidonic acid (AA) metabolites and 5-LO pathway promote angiogenesis and carcinogenesis [43,44,45]. In the present study, the LTB_4_ level was not increased by VEGF stimulation, and this agreed with a previous report which demonstrated that VEGF had no effect on the synthesis of LTB_4_ and 5-LO [43]. In addition, we did not detect any accumulation of Cys-LTs such as LTC_4_, LTD_4_, and LTE_4_ with the stimulation of VEGF (data not shown), although, it was elucidated that exogenous LTD_4_/LTE_4_ signaling via its BLT/CysLT receptors triggers angiogenic response [46,47]. Throughout our experiments, zileuton inhibited several features of VEGF-induced angiogenesis both in vitro and in vivo, including proliferation, migration, and tube formation. Unfortunately, we do not know how zileuton has anti-angiogenic functions without LTB_4_ inhibition in VEGF-induced angiogenic model at this point. Therefore, it is reasonable to speculate that other mechanisms than 5-LO inhibition might contribute to the anti-angiogenic action of zileuton. It is well known that cyclooxygenase enzyme COX-1 and COX-2 have been shown to play an important role in the regulation of angiogenesis [48]. Several studies have been performed that exogenous VEGF in endothelial cells, acting through VEGFR2, upregulated COX-2 with a corresponding increase in PGI_2_ [49]. In similar studies, VEGF increased PGI_2_ via the VEGFR1–VEGFR2 heterodimer and upregulated COX-2 via the PLCγ-IP_3_/Ca^2+^-calcineurin-NFAT pathway [50,51].

One of the interesting findings emerging from the previous study was the apparent inhibitory effect of zileuton on the production of prostanoids. Several reports suggested that genetic (5-LO gene knockout) or pharmacological inhibition of 5-LO by zileuton in murine peritoneal macrophages activated with lipopolysaccharide/interferon-γ (LPS/IFN-γ) led to a critical decrease in the production of prostanoids (i.e., PGE_2_ and PGI_2_) [52,53]. According to these reports, one of possible mechanism by zileuton could be related to the modulation of prostanoids. Therefore, we investigated the effect of zileuton on the regulation of prostanoids in our VEGF-induced angiogenic system. In the present study, VEGF treatment alone strongly augmented PGI_2_ production, which was prevented by cotreatment with zileuton. Conversely, VEGF treatment did not exhibit significant effects on the PGE_2_ production. These observations were substantiated using COX inhibitor indomethacin. Treatment with indomethacin fully abrogated VEGF-induced PGI_2_ production. Taken together, the present findings suggest that zileuton may have its antiangiogenic effect through inhibition of prostanoid, especially PGI_2_. However, further evaluations are needed to elucidate the angiogenic action of zileuton in vivo and in vitro. Another possible scenario is that the anti-angiogenic effect of zileuton comes from preventing the 12-LO pathway, not the 5-LO, thereby blocking the production of 12-HETE, which is the metabolite of 12-LO pathway. In fact, a previous study showed that zileuton was effective in inhibiting biosynthesis of multiple AA metabolites, including 12- and 15-hydroxyeicosatetranolic acid (12-, 15-HETE) in hamster cheek pouch carcinogenesis model [54]. Furthermore, it was also reported that 12-HETE is involved in mediating VEGF-induced angiogenesis [41]. Nevertheless, without further empirical evidence, the speculation remains a speculation requiring further studies on the pharmacological and biological functions of zileuton in more details. As alternatives, we focused on the role of the BK channel as a possible modulator in the present study.

BK channels are found in the plasma membrane of the majority of mammalian cells. These channels play a critical regulatory role in cellular functions and signaling by maintaining the resting membrane potential of cells, making them an effective target for therapeutic control [55,56,57]. In fact, a previous report has demonstrated that activation of BK channel could inhibit cell proliferation and induce apoptosis in EC [19]. Moreover, augmented outwards K^+^ currents by NS1619, a specific BK channel opener, has been shown to induce apoptosis in ovarian cancer cells [18]. Taken together, pharmacological activation of BK channels would be expected to prevent angiogenesis by inducing apoptosis. Thus, we postulated that BK channel might be responsible for the anti-angiogenic and pro-apoptotic actions of zileuton. Initially, we provided physiologic data that zileuton can modulate the amplitude of whole-cell K^+^ currents at all voltages from −60 to +60 mV using whole-cell patch clamp recording. The results showed that 50 µM zileuton increased K^+^ current by about three-fold, and the same concentration of zileuton abrogated the angiogenic effects of VEGF. These finding suggested that increased outward K^+^ currents may contribute to the anti-angiogenic action of zileuton on ECs. We also observed that anti-angiogenic effects of zileuton were abolished by pretreatment with IBTX, a BK channel blocker. Further, knockdown of BK channel expression by siRNA also abolished the anti-angiogenic effect of zileuton, implying that zileuton induces anti-angiogenic effects by BK channel opening either directly or indirectly. Additionally, treatment with NS1619 exhibited anti-angiogenic effects, which were reversed by IBTX. Our data also showed that zileuton or NS1619 trigger apoptosis in the VEGF-induced angiogenesis, this was attenuated by IBTX, suggesting that K^+^ efflux after BK channel opening may induce apoptosis. When K^+^ efflux is increased, intracellular Ca^2+^ concentration is augmented in ECs. Therefore, it was necessary to investigate whether zileuton influences the intracellular Ca^2+^ levels. As we have shown in the present study, zileuton and NS1619 increased intracellular Ca^2+^ levels in early time, which was markedly augmented later when either zileuton or NS1619 were co-treated with VEGF. Thus, these results suggest that antiangiogenic action of zileuton is due to activation of BK channels and the resultant cytosolic K^+^ loss through opened BK channels is able to trigger for apoptosis in EC. Together, our present results strongly substantiate our original conjecture that BK channels are important mediators of the antiangiogenic effect of zileuton.

VCAM-1 and ICAM-1 are induced in disease status including inflammation, atherosclerosis, vascular injury, and angiogenesis [58]. It is widely believed that relevance of integrins α4β1 (VLA-4)/VCAM-1 and α_L_β2 (LFA-1)/ICAM-1 play a key role in angiogenesis. Moreover, much attention has been paid to the interaction between VCAM-1/α4β1 integrin. For example, it was observed that VCAM-1/ α4β1 integrin were individually expressed on vascular smooth muscle cells and ECs in the developing vessels of breast cancer, and found that the administration of an anti-murine VCAM-1 antibody (M/K-2) specifically reduced microvessel formation in Matrigel plug mouse models [59]. More recently, Ig-like domain 6 of VCAM-1 (VCAM-1-D6) was identified as a potential angiogenic target [60]. Thus, targeting VCAM-1 may be an effective strategy for regulating tumor angiogenesis. In the present study, we observed that VCAM-1 and ICAM-1 were induced by VEGF in our experimental system, and this was abrogated by zileuton. This suggests that zileuton may modulate the adhesion molecules-dependent angiogenic processes in ECs.

Accumulating evidences have revealed that efficient angiogenesis requires bioactive NO synthesized by eNOS, which is expressed predominantly in vascular ECs. It has been reported that NO is an important regulator of VEGF-induced angiogenesis that can be prevented by L-NAME [61]. Indeed, our study found that increased NO production and eNOS phosphorylation by VEGF were attenuated by zileuton treatment in ECs. ETS transcription factor Erg is found in quiescent and proliferation in ECs and plays an important role in angiogenesis regulation [62]. Some reports have implicated a role for Erg in the regulation of EC function during inflammation. For example, Erg expression is down regulated upon inflammatory stimulation both in vivo and in vitro [63]. Erg can function as a transcriptional repressor of selected pro-inflammatory gene such as IL-8 and ICAM-1, which promote the recruitment of neutrophils to endothelium [64]. In fact, Erg inhibits pro-inflammatory signaling pathway by inhibiting NF-κΒ activation in ECs. Although it has long been proposed that Erg has broad anti-inflammatory actions in ECs, an opposite evidence of Erg action has been observed. This report showed that inhibition of Erg in ECs results in a dramatic decrease in neovascularization of the plug through the loss of adhesion molecule vascular endothelial (VE)-cadherin, providing evidence for angiogenic regulation of Erg. Moreover, Erg inhibition also resulted in increased cell death, indicating that Erg regulates endothelial survival. They further assumed that Erg may also play a role in the regulation of Bcl-2. Thus, we evaluated whether an Erg transcription factor is involved in zileuton-induced antiangiogenic activity. Based on our results, zileuton inhibited VEGF-induced Erg expression. Studies on the relationship between Erg and apoptotic gene in zileuton action during angiogenesis are underway.

## 5. Conclusions

Our results demonstrate that zileuton inhibits PGI_2_ production and further may regulate angiogenesis via BK channel opening, and this effect is mediated through induction of apoptosis. Moreover, adhesion molecules such as VCAM-1 and ICAM-1 pathway play an important role in the antiangiogenic action of zileuton. Thus, these results provide new insight into the mechanisms responsible for the activity of zileuton and suggest its clinical potential as a treatment for diseases involving angiogenesis, such as cancers.

## Supplementary Materials

The following are available online at https://www.mdpi.com/2073-4409/8/10/1182/s1, Figure S1: Effects of zileuton on VEGF-induced PGI2 production in HUVECs.
