# Supplementary material for: Zileuton, a 5-Lipoxygenase Inhibitor, Exerts Anti-Angiogenic Effect by Inducing Apoptosis of HUVEC via BK Channel Activation
Source: Cells. 2019 Sep 30;8(10):1182. doi: 10.3390/cells8101182 (PMC6829222; doi:10.3390/cells8101182)
Supplement: Supplementary file 1 [file cells-08-01182-s001.pdf]

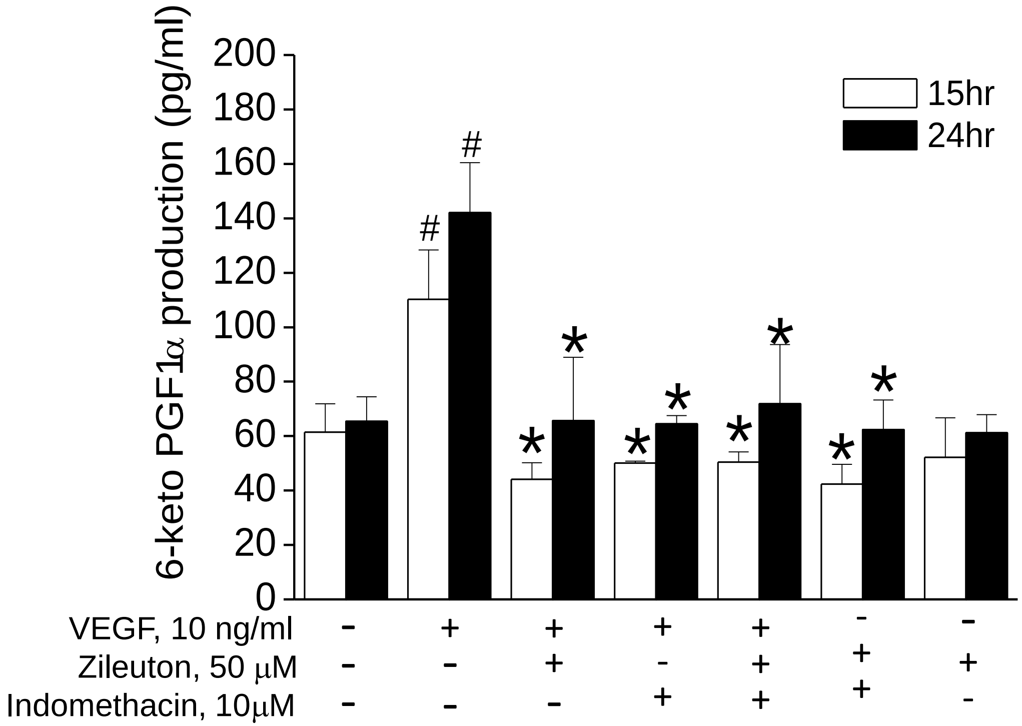

Figure S1. Effects of zileuton on VEGF-induced PGI_2 production in HUVECs. HUVECs were incubated with either zileuton (50 μM) or indomethacin (10 μM) for 1 h and then stimulated with VEGF (10 ng/mL) for 15 or 24 h. 6-keto PGF1α production was determined by Elisa kits. Data shown represent the means ± SD from three independent experiments (each performed in duplicate). $^{\#}p < 0.05$ versus non-treated cells, $^{*}p < 0.05$ versus VEGF-treated cells.

Supplementary Figure
